# Predicting haemodialysis arteriovenous fistula outcomes using computational fluid dynamics and ferumoxytol-enhanced MRI

**DOI:** 10.1177/11297298251395144

**Published:** 2025-12-07

**Authors:** Robert Ker, George Hyde-Linaker, Pauline Hall Barrientos, David Brian Kingsmore, Sokratis Stoumpos, Asimina Kazakidi

**Affiliations:** 1 Renal and Transplant Unit, Queen Elizabeth University Hospital, Glasgow, UK; 2 Department of Biomedical Engineering, University of Strathclyde, Glasgow, UK; 3 Imaging Centre of Excellence, Queen Elizabeth University Hospital, Glasgow, UK; 4 School of Cardiovascular & Metabolic Health, University of Glasgow, Glasgow, UK

**Keywords:** Arteriovenous fistula, haemodynamics, chronic kidney disease, computational fluid dynamics, ferumoxytol, MRI

## Abstract

**Objective:**

Arteriovenous fistulas (AVF) are the preferred vascular access for maintenance haemodialysis. However, AVF non-maturation occurs in up to 60% of patients, frequently caused by inadequate vascular remodelling or stenosis development. This study explores the relationships between AVF anatomy, haemodynamics and AVF outcomes by combining, uniquely, high-fidelity numerical simulations and state-of-the-art ferumoxytol-enhanced magnetic resonance imaging (Fe-MRI) in patients.

**Methods:**

Patients underwent Fe-MRI 6 weeks after AVF creation. A novel computational fluid dynamics (CFD) methodology was employed to rigorously investigate haemodynamic metrics, including wall shear stress (WSS) and oscillatory shear index (OSI), and quantify changes in the AVF lumen at 1 cm intervals along the proximal artery, anastomosis and AVF vein. The primary outcome was AVF success, defined as AVF usage (assisted or unassisted) for dialysis for at least 3 months. ROC analysis was conducted to assess anatomical predictors of AVF flows of ⩾1000 ml/min.

**Results:**

The analysis included 17 AVFs (13 successful, 4 failed). Compared to failed fistulas, successful AVFs had higher mean WSS and OSI. Failed AVFs exhibited different haemodynamics, including lower flow rates with less helical flow. On ROC analysis, the three metrics associated with the highest area under the curve (AUC) values were the feeding artery curvature (0.82) and diameter (0.76), and draining vein diameter (0.74), with a combined AUC value of 0.83.

**Conclusion:**

These data suggest that high WSS, OSI, larger feeding artery and draining vein diameters and lower feeding artery curvature are associated with successful AVF outcomes. Whilst venous parameters are important, this study highlights the critical role of feeding artery characteristics, particularly diameter and curvature. These findings provide significant insights into the role of haemodynamics and geometry in modulating AVF maturation, suggesting that incorporation of arterial metrics into preoperative assessments could enhance surgical decision-making for more reliable AVF maturation and better long-term outcomes in haemodialysis patients.

## Introduction

Arteriovenous fistulas (AVF) are the preferred vascular access for haemodialysis.^1,2^ AVF non-maturation is common, occurring in up to 60% of patients,^2–5^ while 50% of patients require at least one further procedure to facilitate maturation.^6,7^ Non-maturation is commonly associated with anatomic problems of the AVF,^1,8,9^ principally inadequate vascular remodelling and stenosis triggered by neointimal hyperplasia.^9,10^ Surgical training and technique can improve AVF and patient outcomes independent of vessel or patient characteristics.^11,12^

To better understand the mechanisms of vascular remodelling, blood flow dynamics throughout the AVF need to be delineated. Wall shear stress (WSS) is the vascular luminal surface friction exerted by flowing blood and is a strong local regulator of vascular remodelling.^13^ WSS varies over time and location, due to the pulsatile blood flow and geometric irregularities of the vessel wall,^14^ being particularly patchy around branching ostia.^15,16^ Oscillatory shear index (OSI) represents the variation in flow direction. OSI is unitless and ranges from 0 (unidirectional flow) to 0.5 (flow without predominant mean shear direction). A recent study suggested that high WSS promotes, but high OSI inhibits, lumen expansion after AVF creation in both the feeding artery and draining vein.^14^

Computational fluid dynamics (CFD) can accurately measure haemodynamic factors, which are difficult to measure in vivo. CFD models generate a detailed analysis of patient-specific anatomical characteristics, identify features associated with abnormal flow^17,18^ and predict fistula maturation using flow descriptors.^19^

Imaging of AVF is used to assess vessel characteristics, flow rates and identify anatomical issues such as stenosis. Ferumoxytol, an iron oxide nanoparticle, provides a safe alternative to gadolinium contrast for MR angiography in CKD.^19^ Ferumoxytol-enhanced magnetic resonance imaging (Fe-MRI) is superior to traditional imaging techniques for vascular mapping by effectively identifying central venous stenosis, and characterising vessel size, patency, course and tortuosity.^20^

In this prospective observational study, we aimed to explore the relationships between AVF anatomical parameters, haemodynamics and AVF outcomes by using CFD simulations and high-quality images obtained with Fe-MRI.

## Methods

Fifty-one patients underwent vascular mapping by Fe-MRI 6 weeks after AVF creation in a prospective study conducted from 2016 to 2018.^20^ The study was approved by the institutional review board (Research Ethics Committee reference number, 16/NS/0099) and registered with ClinicalTrials.gov (NCT02997046). Funding was provided by the Glasgow Renal and Transplant Unit endowment fund, and ferumoxytol was supplied by AMAG Pharmaceuticals free of charge.

Inclusion criteria were patients >18 years old with CKD requiring vascular mapping before AVF creation. Exclusion criteria included contraindications to MRI, history of allergic reaction to intravenous iron, iron overload and multiple or serious co-morbidities (such as active cancer or dementia). Both incident and prevalent dialysis patients were included, with previous arteriovenous accesses or central venous catheterisation.^21^ Written informed consent was obtained from all patients. All scans were performed on a 3.0-T Prisma MRI scanner (Magnetom; Siemens Healthineers, Erlangen, Germany) with local phased-array imaging coils using a standardised protocol, with patients in the supine position.

Of these 51 participants who underwent study-specific mapping protocols followed by AVF creation in the original study,^21^ 17 patients were selected for CFD modelling to further evaluate the specific AVF anatomy and haemodynamics. The selection was random after review of the image quality and AVF outcomes were not known at the time of selection. All included fistulas had end-to-side anastomoses, which is the preferred configuration in our centre as it preserves the vessel’s natural flow, is technically less demanding and has a lower risk of complications like arterial steal syndrome.^22^

**Figure 1.**
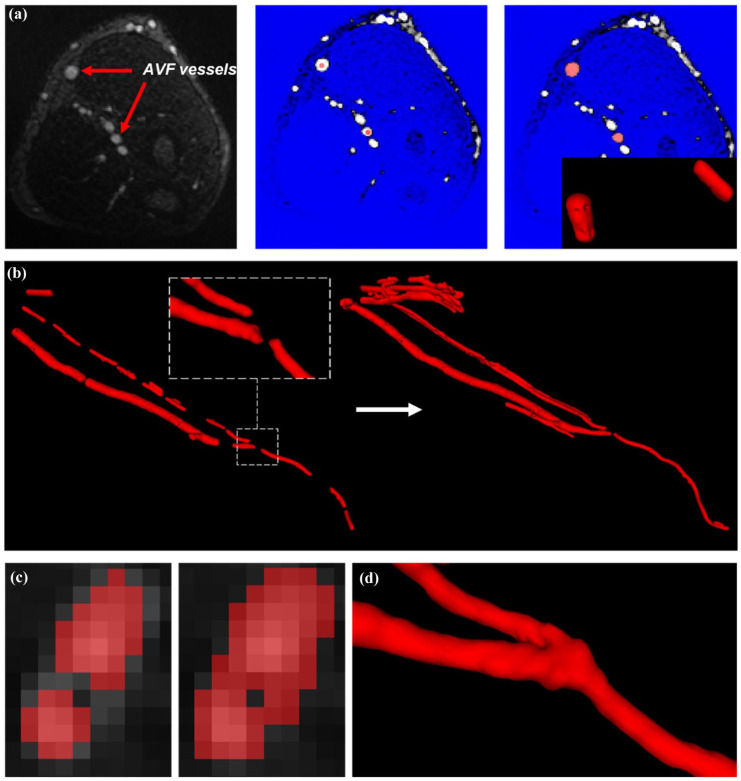
Example of AVF vessel segmentation using ITK-SNAP: (a) the process of applying the ‘snake-evolution’ tool in the 2D view, where the blue region represents the excluded greyscale values, (b) evolving segmentations using the tool, (c) manual segmentation at the AVF anastomosis and (d) resulting segmentation with Gaussian smoothing in ITK-SNAP.

The workflow used to convert Fe-MRI images into three-dimensional computational models was detailed in a recently published work from our team.^21^ The images obtained from the Fe-MRI were processed using ITK-SNAP (www.itksnap.org, Figure 1(a)–(d)) to generate a mesh of the surface of the AVF, which was verified against the location of the vessel lumen on the original Fe-MRI images. The anatomically correct model of the AVF allowed for detailed measurement of anatomical parameters: anastomosis angle, feeding artery diameter and curvature, and draining vein diameter and curvature.

The inlet and outlets of the CFD computational domain were considered at locations where the flow rates were known from phase-contrast MRI (PC-MRI) measurements.^21^ The AVF vessels were further subdivided into 1 cm segments to allow a thorough investigation of individual localised haemodynamics. The anastomosis area was kept separate from other subdivisions as this was an area of particular interest.^22^

A scale-resolving hybrid turbulence model in STAR-CCM+ was used to simulate blood flow within individual AVFs.^21^ CFD simulations measured several haemodynamic metrics associated with WSS, that is, time-averaged wall shear stress (TAWSS), wall shear stress spatial gradient (WSSG), velocity streamlines and localised normalised helicity (LNH) captured at the peak systole of the cardiac cycle of each patient. These haemodynamic metrics are defined in the Supplemental Materials. This allowed us to establish the influence of AVF geometry and haemodynamics on AVF outcomes.

The primary outcome was AVF success, defined as AVF use (assisted or unassisted) for haemodialysis for a minimum of 3 months. In addition, ROC analysis assessed anatomical predictors of high venous flows. AVFs were subdivided into high venous flow (⩾1000 ml/min) versus lower flow (<1000 ml/min). This cut-off is higher than the traditional target of >600 ml/min, but was chosen as although flows of 600–1499 ml/min are effective for dialysis,^23^ AVFs with flows of >1000 ml/min are unlikely to have stenosis^24^ and are associated with better outcomes, with lower all-cause mortality.^25^ ROC analysis to model the performance of 11 candidate parameters (7 AVF, and 4 patient-specific variables) on high venous flows was performed using the R Core Team software.

## Results

Of the 17 patients included in the study, 13 had successful AVFs and 4 had AVFs that failed to mature or were used for less than 3 months prior to failure. There were 10 radio-cephalic fistulas (RCF), 6 brachio-cephalic fistulas (BCF) and 1 brachio-basilic fistula (BBF). Fifteen fistulas were left-sided and two right-sided. Patient demographics are provided in Table 1.

**Table 1.** Patient demographics.

Patient demographic	Value
Age (years), mean (SD)	58 (15.1)
Sex, *n* (%)	
Men	11 (64.7)
Women	6 (35.2)
Race, *n* (%)	
White	13 (76.5)
Asian	4 (23.5)
Body mass index categories, *n* (%)	
<18.5	1 (5.9)
18.5–24.9	5 (29.4)
25–30	5 (29.4)
>30	6 (35.3)
Cause of end-stage kidney disease, *n* (%)	
Diabetes	4 (23.5)
IgA nephropathy	2 (11.8)
Pyelonephritis	2 (11.8)
Unknown	2 (11.8)
Other^a^	7 (41.2)
Smoking status, *n* (%)	
Never smoked	10 (58.8)
Ex-smoker	6 (35.3)
Current smoker	1 (5.9)
KRT prevalence, *n* (%)	
Low clearance	12 (70.6)
Established on haemodialysis	4 (23.5)
Transplant (failing)	1 (5.9)
At least one previous arteriovenous access, *n* (%)	2 (11.8)
At least one previous central venous catheterisation, *n* (%)	4 (23.5)

a Congenital dysplasia (*n* = 1), cyclosporine toxicity (*n* = 1), hypertension (*n* = 1), ischaemic nephropathy (*n* = 1), obstructive uropathy (*n* = 1), polycystic kidney disease (*n* = 1), renovascular disease (*n* = 1).

The measured anatomical parameters are summarised in Table 2. Successful AVFs had a greater feeding artery diameter (4.71 ± 1.45 mm vs 4.18 ± 1.70 mm, *p* = 0.323), and smaller feeding artery curvature (0.030 ± 0.014 m^−1^ vs 0.042 ± 0.017 m^−1^, *p* = 0.167) compared to unsuccessful AVFs. Draining vein diameter and curvature were greater in successful AVFs (6.48 ± 1.53 mm vs 6.04 ± 2.17 mm, *p* = 0.379 and 0.038 ± 0.010 m^−1^ vs 0.055 ± 0.013 m^−1^, *p* = 0.056, respectively). The mean difference in size between the draining vein and feeding artery was similar in successful compared to unsuccessful AVFs (1.77 ± 1.32 mm vs 1.86 ± 1.22 mm, *p* = 0.458). The mean anastomosis angle was tighter in successful than unsuccessful AVFs (61.6° ± 20.9° vs 75.8° ± 13.1°, *p* = 0.09).

**Table 2.** Subject population centreline characteristics (where mean feeding artery and draining vein characteristics were evaluated up to 10 cm along centreline length, and draining artery characteristics were evaluated up to 5 cm along centreline length).

Patient number	Fistula type	Long term outcome	Venous outflow average (ml/min)	Venous outflow category	Feeding artery diameter (mm)	Feeding artery curvature (m^−1^)	Draining vein diameter (mm)	Draining vein curvature (m^−1^)	Anastomosis angle (°)
#71	Left BCF	S	1507	High	6.07	0.013	8.06	0.044	64.7
#69	Left BCF	S	1277	High	4.81	0.028	7.94	0.022	75.8
#60	Left BCF	S	1783	High	6.724	0.014	7.9	0.035	80.4
#40	Left BCF	S	1361	High	5.23	0.018	7.18	0.036	91.4
#77	Left RCF	S	862	Low	3.11	0.045	4.63	0.062	24
#65	Left RCF	S	1753	High	3.84	0.021	8.27	0.036	26.1
#87	Left RCF	S	599	Low	2.64	0.036	5.51	0.036	31.5
#06	Left RCF	S	1033	High	5.00	0.024	4.89	0.038	59.4
#57	Left RCF	S	454	Low	4.64	0.045	3.92	0.026	60.1
#18	Left RCF	S	829	Low	4.80	0.025	6.78	0.042	61.6
#64	Left RCF	S	906	Low	2.92	0.045	4.62	0.036	72.2
#34	Left RCF	S	705	Low	3.72	0.06	6.24	0.055	72.9
#45	Right BBF	S	2864	High	7.73	0.02	8.25	0.029	81.1
#44	Left BCF	U	47	Low	2.75	0.046	2.83	0.075	94.9
#79	Left RCF	U	577	Low	2.23	0.035	5.45	0.054	60
#72	Left RCF	U	1368	High	5.83	0.067	7.23	0.055	80.1
#80	Right BCF	U	694	Low	5.92	0.02	8.64	0.037	68.2

L: left arm; R: right arm; BCF: brachiocephalic fistula; RCF: radiocephalic fistula; BBF: brachiobasilic fistula; S: successful AVF outcome; U: unsuccessful AVF outcome.

Eight patients had AVFs with high flow rates (⩾1000 ml/min), and nine had lower flow rates (<1000 ml/min). Mean venous outflow was higher in successful compared to unsuccessful AVFs (1225.4 ± 623.7 ml/min vs 671.3 ± 470.4 ml/min, *p* = 0.07).

Differences were also demonstrated between the different AVF configurations. RCFs had a significantly tighter mean anastomosis angle (54.8° ± 19.2° vs 79.5° ± 10.3°, *p* = 0.003), and higher feeding artery curvature (0.040 ± 0.014 m^−1^ vs 0.023 ± 0.011 m^−1^, *p* = 0.008) than other anatomical types of AVFs. Unsurprisingly, the feeding artery and draining vein diameters were smaller in RCFs (3.87 ± 1.11 mm vs 5.60 ± 1.46 mm, *p* = 0.02 and 5.76 ± 1.29 mm vs 7.26 ± 1.85 mm, *p* = 0.06, respectively). The average venous outflow was lower in RCFs compared to other anatomical types of AVFs (909 ± 374 ml/min vs 1352 ± 814 ml/min, *p* = 0.12).

**Figure 2.**
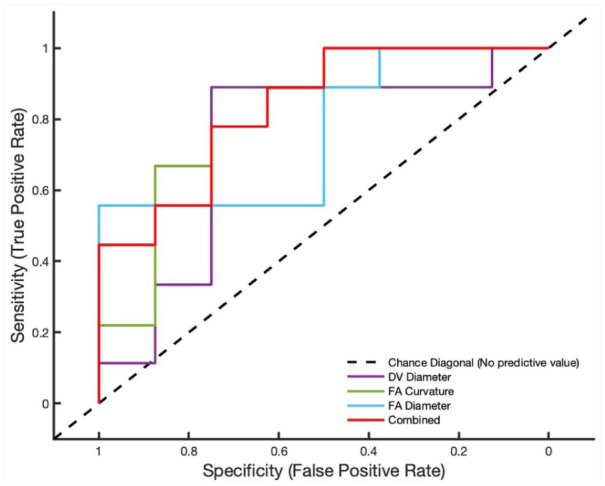
ROC curves for the feeding artery (FA) curvature, feeding artery diameter, draining vein (DV) diameter and the combined ROC curve of the three metrics using original datasets (*N* = 17).

The area under the curve (AUC) for each candidate parameter is shown in Table 3. The three variables with the highest AUC values were the feeding artery curvature, feeding artery diameter and draining vein diameter. ROC analysis of these three candidate parameters combined was performed, resulting in an AUC of 0.83 (Figure 2). This was higher than the AUC of each independent variable and indicates a strong correlation between all three variables and high venous flow AVF.

**Table 3.** Area under curve (AUC) of ROC curve.

Characteristic	AUC (raw data)
Anastomosis angle	0.6667
Feeding artery diameter	0.7639
Draining artery diameter	0.7222
Draining vein diameter	0.7361
Feeding artery curvature	0.8194
Draining artery curvature	0.7014
Draining vein curvature	0.4583
Age	0.6042
Body mass index (BMI)	0.7083
Systolic blood pressure	0.6528
Heart rate	0.7014

**Figure 3.**
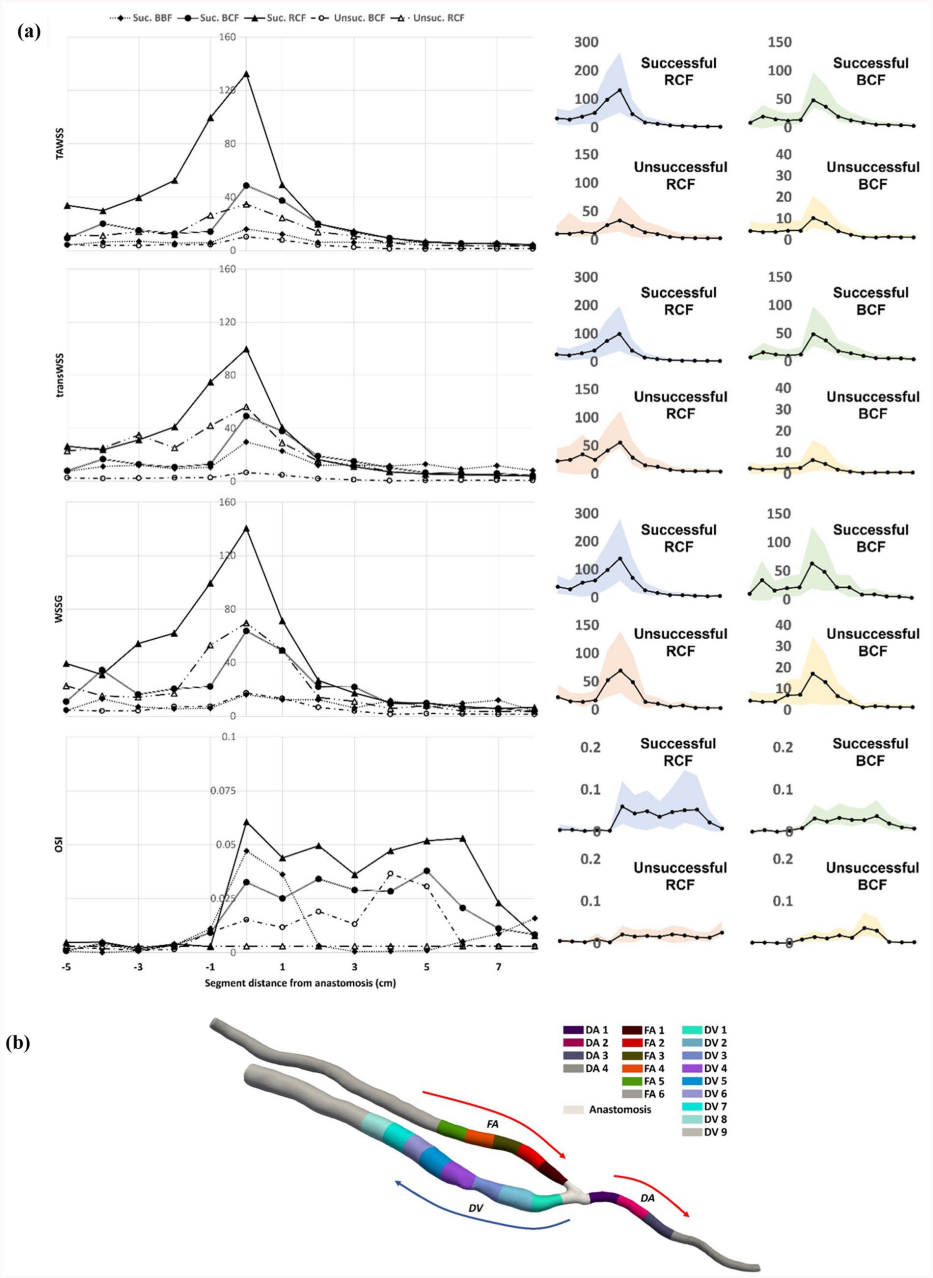
(a) Mean WSS metrics (and error bars (right)) for each sub-group at 1 cm segments for the computational AVF. Note that the segments presented follow the segments along the centreline from the feeding artery and draining vein as shown in (b).

The CFD analysis generated contour plots of TAWSS, WSSG, velocity streamline and LNH at peak systole. Plots comparing successful and unsuccessful AVFs are demonstrated in the Supplemental Figures 1 to 3. Figure 3(a) demonstrates the measured WSS metrics in 1 cm segments along the AVF model (Figure 3(b)), and shows the results subdivided between anatomical fistula types and outcome. Elevated WSS (TAWSS and WSSG) was seen at the juxta-anastomotic vascular segments. Radiocephalic AVFs appeared to have higher WSS than other types of AVF, potentially due to the smaller cross-sectional area of the vessel. Successful AVFs had a larger surface area where high WSS was present. Unsuccessful AVFs had lower flow rates with less helical flow. Low oscillatory shear index (OSI) values were present in the feeding artery, and subsequently rose significantly after the anastomosis, demonstrating turbulent and rotational flow moving into the vein. Higher OSI was seen in the successful compared to the unsuccessful AVFs, and RCFs had higher OSI than BCFs.

## Discussion

We utilised Fe-MRI and CFD simulations to assess the haemodynamics of newly-created AVFs with several noteworthy findings. First, haemodynamic parameters, such as high WSS and OSI were predictive of a successful AVF outcome. Second, the main anatomical parameters promoting high venous outflow included larger feeding artery and draining vein diameters, and lower feeding artery curvature. Optimising vessel selection or surgical technique should promote these favourable anatomical parameters. This provides important insights into the impact of haemodynamics and geometry on AVF maturation.

Higher TAWSS, transWSS and WSSG values corresponded to improved AVF outcomes. WSS is detected by endothelial receptors, stimulating vasodilatation and promoting vascular remodelling by increasing venous diameter, which in turn normalises WSS levels.^19,26^ This increased lumen diameter enables successful repeat cannulation of the AVF.^21,26–28^ The highest levels of WSS were found at the anastomosis. The sutures at the anastomoses may limit lumen expansion at this point, keeping WSS levels high and preventing neointimal hyperplasia development.^27^ This concept is reinforced by a recent RCT that demonstrated the interrupted sutures at the anastomoses led to higher patency rates of RCFs.^29^

An inverse correlation between WSS during AVF creation, and stenosis development post-operatively, has been demonstrated in animal models.^28,30^ Low WSS is associated with the development of cephalic arch stenosis,^31^ and mean WSS has also been demonstrated to correlate with lumen area expansion.^14^ This is in keeping with our study, where successful AVFs had high WSS over a larger surface area. AVFs have significantly higher WSS values compared to physiological levels, due to high pressure from the feeding artery being directly transmitted, and disrupted flows of blood from the anastomosis angle.^14^ Very high WSS can trigger endothelial damage and generate adverse vascular remodelling or even propagate in-vessel thrombosis.^27,32^ Therefore, it is not clear what is the optimum distribution or level of WSS to promote AVF maturation.

OSI levels were low leading up to the anastomosis and significantly increased immediately post-anastomosis, demonstrating highly-disturbed blood flow created by the acute change in blood flow direction. A key difference between the successful and unsuccessful sub-groups was the mean OSI observed in the anastomosis and venous segments, with higher values being observed in the successful cases. This difference was more pronounced in the RCF group than in the BCF group. This may be due to the successful AVFs, in general, having higher venous outflow, with greater velocity, correlating with higher OSI. In contrast to our study, some studies have shown high OSI was negatively associated with AVF lumen expansion.^14^ While lumen expansion is a key factor in AVF maturation, it does not necessarily correlate to AVF success which was the primary endpoint in our study. These alternative endpoints may explain this difference between the studies. The pattern of OSI was the same between studies, with low OSI in the feeding artery, a dramatic increase at the level of anastomoses and sustained elevated OSI in the draining vein. He et al. noted that OSI increased in the draining vein between creation and week 6, and then began to fall at 6 months.^14^ Therefore, the elevated OSI captured at the 6-week Fe-MRI imaging in our study may be reflecting an active process of vascular remodelling, which is key to AVF development.

WSS and OSI are directly influenced by vessel geometry and size. In particular, larger cross-sectional anastomotic areas have been associated with higher blood flow through the fistula and less pressure drop across the anastomosis. However, this did come with increased risk of distal ischaemia due to reversed arterial outflow.^33,34^ This could be explained in simplified terms using the haemodynamic analogy of Ohm’s and Hagen-Poiseuille’s laws. Increasing the anastomotic area, reduces the vascular resistance locally, increasing the flow rate Q for a specific pressure gradient or reducing the pressure difference for a given Q. For very large cross-sectional anastomoses, however, the venous resistance may become so low that the pressure at the anastomotic region becomes less than the pressure in the distal artery (P_feeding artery_ > P_distal artery_ > P_anastomosis_ ≈ P_vein_), causing the reversal of flow from the downstream artery back towards the anastomosis, with a risk of distal ischaemia, and increased pressure gradient ΔP = P_feeding artery_ − P_anastomosis_ across the anastomosis. Thus, for a successful anastomosis, the pressure distribution should be maintained such that P_feeding artery_ > P_distal artery_ ≈ P_anastomosis_ > P_vein_, with a beneficial smaller pressure drop ΔP = P_feeding artery_ − P_anastomosis_.

Turbulent blood flow can create high-frequency vibration within the vessel wall, which may contribute to the development of intimal hyperplasia by the disruption of normal cell signalling pathways.^35^ However, that work examined the effect of high-frequency vibration within arteries and smooth muscle cells^35^, rather than veins where, in our study, high OSI and turbulent flow were seen.^[Bibr bibr35-11297298251395144]^

In this study, successful AVF had a tighter anastomotic angle. Previous studies have shown anastomotic angles of around 30° to be optimal as this angle was associated with reduced OSI, smaller areas of low WSS^36^ and the avoidance of pathologically high WSS.^37^ Artificial devices that fix the anastomotic angle between 40° and 50° have been demonstrated to increase successful fistula outcomes.^38,39^ Similarly, it has been observed that when the anastomotic angle exceeded 58°, the venous outflow reduced significantly and there was arterial backflow.^33^ Lower anastomosis angles (<43°) resulted in less pressure drop in the draining vein, preserving higher levels of WSS.^33^

Minimum ultrasound criteria have been established for AVF maturation at 4 weeks using vessel diameter and flow parameters (AVF vessel diameter 4–5 mm and blood flow of 400–500 ml/min).^1^ Previous guidelines suggest that AVF should have a flow of approximately 600 ml/min, a minimal diameter of 6 mm and lie less than 0.6 cm below the surface of the skin by 6 weeks post-creation (referred to as the ‘rule of six’).^40^ AVFs meeting these criteria, have been shown to be 10 times more likely to support dialysis.^41^ Venous flow and vessel depth have been suggested to be more predictive of AVF maturation and successful use, than the draining vein diameter.^41^ The Fistula Maturation Study also found that AVF blood flow, vein diameter and vein depth at 6 weeks were reliable predictors of AVF outcome,^42^ but did not support the use of specific cut-offs and suggests a patient - specific probability of AVF maturation to be calculated.

Although not seen in this study, venous curvature has been demonstrated to help promote lumen expansion.^27^ He et al. demonstrated AVFs with greater venous curvature required fewer interventions in the first year post creation.^43^ Less attention has been paid in the literature to the impact of arterial curvature, which we have shown to significantly impact levels of venous outflow. Here, there was a significant relationship between reduced arterial curvature and venous outflow. However, the RADAR technique, where the radial artery is divided and anastomosed onto the feeding vein, augments the arterial curvature and has shown to have favourable haemodynamics and better AVF outcomes.^12,44,45^ This difference may be accounted as the majority of the AVFs with lower venous flow were RCFs, as expected given the smaller vessels involved, and RCFs had statistically significantly higher levels of feeding artery curvature (*p* = 0.015), likely due to the implementation of the RADAR technique.

Traditionally, there has been a focus on venous parameters for AVF success. However, Farrington et al. suggested that arterial diameter was of more significance than venous diameters in determining AVF success,^3^ with diameters above 5 mm having greater rates of success compared to those of 3 mm or below.^3^

Arterial inflow has been argued to be the primary force behind vascular remodelling and AVF maturation^3,46^ as it generates the high flow of blood and WSS. While there is a minimum recommended cut off of 2 mm for arterial diameter in AVF creation^1^ as vessels below this threshold cannot generate the intended venous blood flow volumes,^46^ a greater emphasis should be placed on selecting the larger of the available arteries to optimise AVF success. Following the results of our study, the feeding artery curvature should also have a greater role in pre-surgery planning. The combination of arterial factors provides key information for informing the AVF site selection, a process traditionally largely determined by surgeon experience and preference.

Surgical training plays a critical role in ensuring higher success rates for AVF, beyond vessel selection alone. Strengthening training skills by increasing surgical competency can improve patient outcomes and long-term patency of vascular access.^11^

### Limitations

The small cohort size and the small number of patients in each sub-group did not allow analyses of the different AVF anatomical subtypes, which would be useful in the context of the significantly different flow rates and vessel characteristics. However, this was beyond the scope of this pilot study. We only examined AVFs at a single point in time and did not evaluate how the geometry and haemodynamics evolved over time and the impact this may have had on AVF outcomes. Previous work suggested that earlier assessment of AVF at 2 weeks post-surgery was more predictive of AVF outcome than at 6 weeks.^5^ This study was purely mechanistic and did not examine the impact of peri-operative technical factors, patient characteristics or co-morbidities. Diabetes,^2,14^ previous vein injury,^46^ pre-existing vascular disease,^2,47,48^ blood pressure^2,4^ and left ventricular systolic function^3^ have all been suggested as predictors of AVF maturation and success. Chronic uraemia can impair endothelial function,^14^ and therefore the length of time with CKD or requiring haemodialysis^2^ may also impact the success of AVF and this was not examined in this study.

In this investigation, the effect of the length or cross-sectional area of the anastomosis, especially in proximal fistulas, on AVF flow and maturation was not considered, however, these have been shown to impact blood flow and AVF outcome.^33,34^

Lastly, we assumed a rigid vascular wall model, similar to recent studies.^14,49^ Distended veins have limited compliance, whereas the arterial wall has elasticity to account for the pulsatile flow of blood, and therefore this assumption may better evaluate the venous segment but may misrepresent the arterial segment.

## Conclusions

The current practice of pre-operative ultrasound mapping of vessels has not yet led to an improvement in AVF outcomes.^3,50^ By combining Fe-MRI and CFD we have shown that high WSS, high OSI, greater feeding artery and draining vein diameters and lower feeding artery curvature promote successful AVF outcomes. Our findings suggest that greater importance should be placed on the selection of the feeding artery in the planning and peri-operative stages of AVF creation. Further studies on interactions between local and systemic factors and individual patient characteristics in AVF remodelling could provide novel strategies to promote AVF maturation.

## Supplemental Material

sj-pdf-1-jva-10.1177_11297298251395144 – Supplemental material for Predicting haemodialysis arteriovenous fistula outcomes using computational fluid dynamics and ferumoxytol-enhanced MRI Supplemental material, sj-pdf-1-jva-10.1177_11297298251395144 for Predicting haemodialysis arteriovenous fistula outcomes using computational fluid dynamics and ferumoxytol-enhanced MRI by Robert Ker, George Hyde-Linaker, Pauline Hall Barrientos, David Brian Kingsmore, Sokratis Stoumpos and Asimina Kazakidi in The Journal of Vascular Access
