# Supplementary material for: Predicting haemodialysis arteriovenous fistula outcomes using computational fluid dynamics and ferumoxytol-enhanced MRI
Source: J Vasc Access. 2025 Dec 7;27(3):1219–28. doi: 10.1177/11297298251395144 (PMC13135645; doi:10.1177/11297298251395144)
Supplement: sj-pdf-1-jva-10.1177_11297298251395144 – Supplemental material for Predicting haemodialysis arteriovenous fistula outcomes using computational fluid dynamics and ferumoxytol-enhanced MRI [file sj-pdf-1-jva-10.1177_11297298251395144.pdf]

Supplementary Material

Supplementary Table 1. Definitions and abbreviations of different wall shear strain metrics.

**Measured metric**	**Abbreviation and mathematical definition**	**Definition**
Instantaneous wall shear stress (WSS) vector	$\vec{\tau}_\omega$	Instantaneous tangential force induced on the vessel wall by the flowing blood.
Mean WSS	$\vec{\tau}_{mean} = \frac{1}{T}\int_0^T \vec{\tau}_\omega \, dt$	Mean tangential force over a cardiac cycle of period, T.
Time-averaged wall shear stress	$\text{TAWSS} = \frac{1}{T}\int_0^T \lvert\vec{\tau}_\omega\rvert \, dt$	Tangential force induced on the vessel wall averaged over the entire cardiac cycle.
Oscillatory shear index	$\text{OSI} = \frac{1}{2}\left(1 - \frac{\lvert\int_0^T \vec{\tau}_\omega dt\rvert}{\int_0^T \lvert\vec{\tau}_\omega\rvert dt}\right) = \frac{1}{2}\left(1 - \frac{\lvert\vec{\tau}_{mean}\rvert}{\text{TAWSS}}\right)$	Measurement of the variation in the flow directionality. Unitless, ranging from 0 (unidirectional flow) to 0.5 (flow with no mean predominant direction).
Transverse wall shear stress	$\text{transWSS} = \frac{1}{T}\int_0^T \left\lvert\vec{\tau}_\omega \cdot \left(\vec{n} \times \frac{\int_0^T \vec{\tau}_\omega dt}{\lvert\int_0^T \vec{\tau}_\omega dt\rvert}\right)\right\rvert dt$ $= \frac{1}{T}\int_0^T \left\lvert\vec{\tau}_\omega \cdot \left(\vec{n} \times \frac{\vec{\tau}_{mean}}{\lvert\vec{\tau}_{mean}\rvert}\right)\right\rvert dt$	Wall shear stress elements perpendicular to the temporal mean WSS vector, averaged over the cardiac cycle.
Wall shear stress spatial gradient	WSSG= $\sqrt{\left(\left\lvert\frac{\partial\vec{\tau}_\omega}{\partial x}\right\rvert\right)^2 + \left(\left\lvert\frac{\partial\vec{\tau}_\omega}{\partial y}\right\rvert\right)^2 + \left(\left\lvert\frac{\partial\vec{\tau}_\omega}{\partial z}\right\rvert\right)^2}$	The magnitude of the variation of WSS over a given area.
Localised normalised helicity	$\text{LNH} = \frac{u \cdot \omega}{\lvert u\rvert \lvert\omega\rvert}$	Measures the alignment between the velocity, u, and the vorticity, ω, vectors.

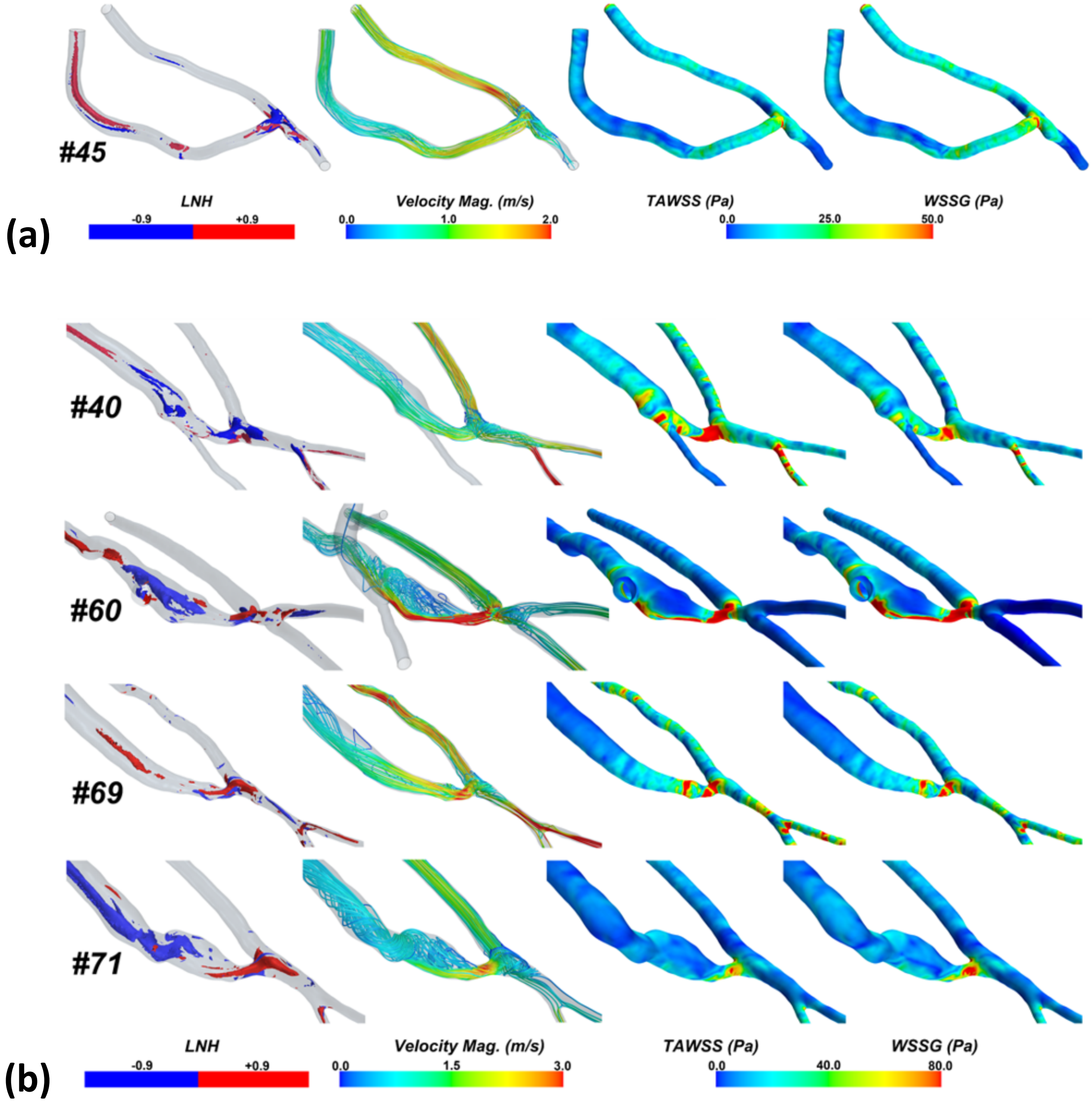

Supplementary Figure 1. NLH and flow streamlines (at peak systole), TAWSS and WWSG contour plots for successful (a) BBF and (b) BCFs.

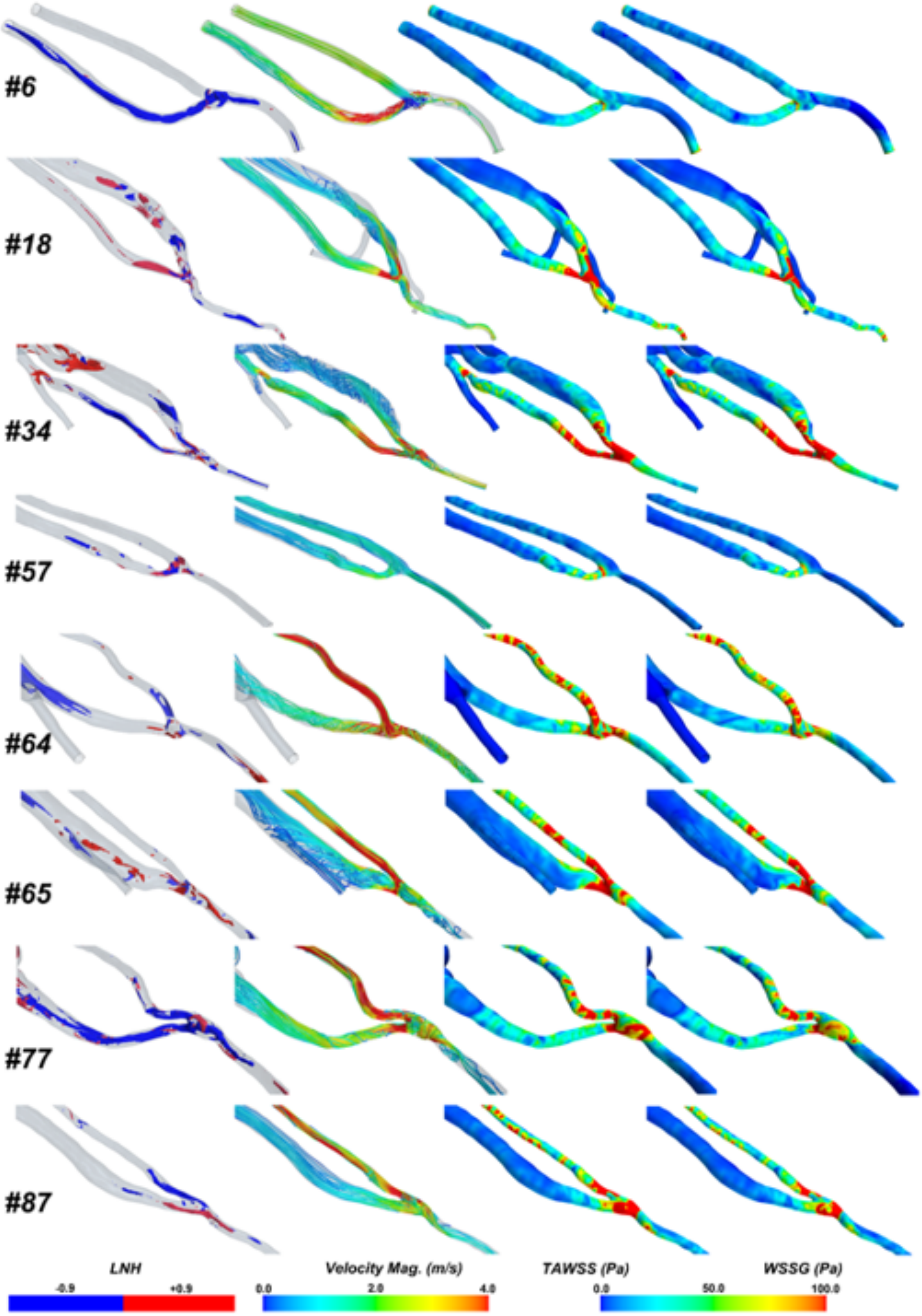

Supplementary Figure 2. NLH and flow streamlines (at peak systole), TAWSS and WWSG contour plots for successful RCFs.

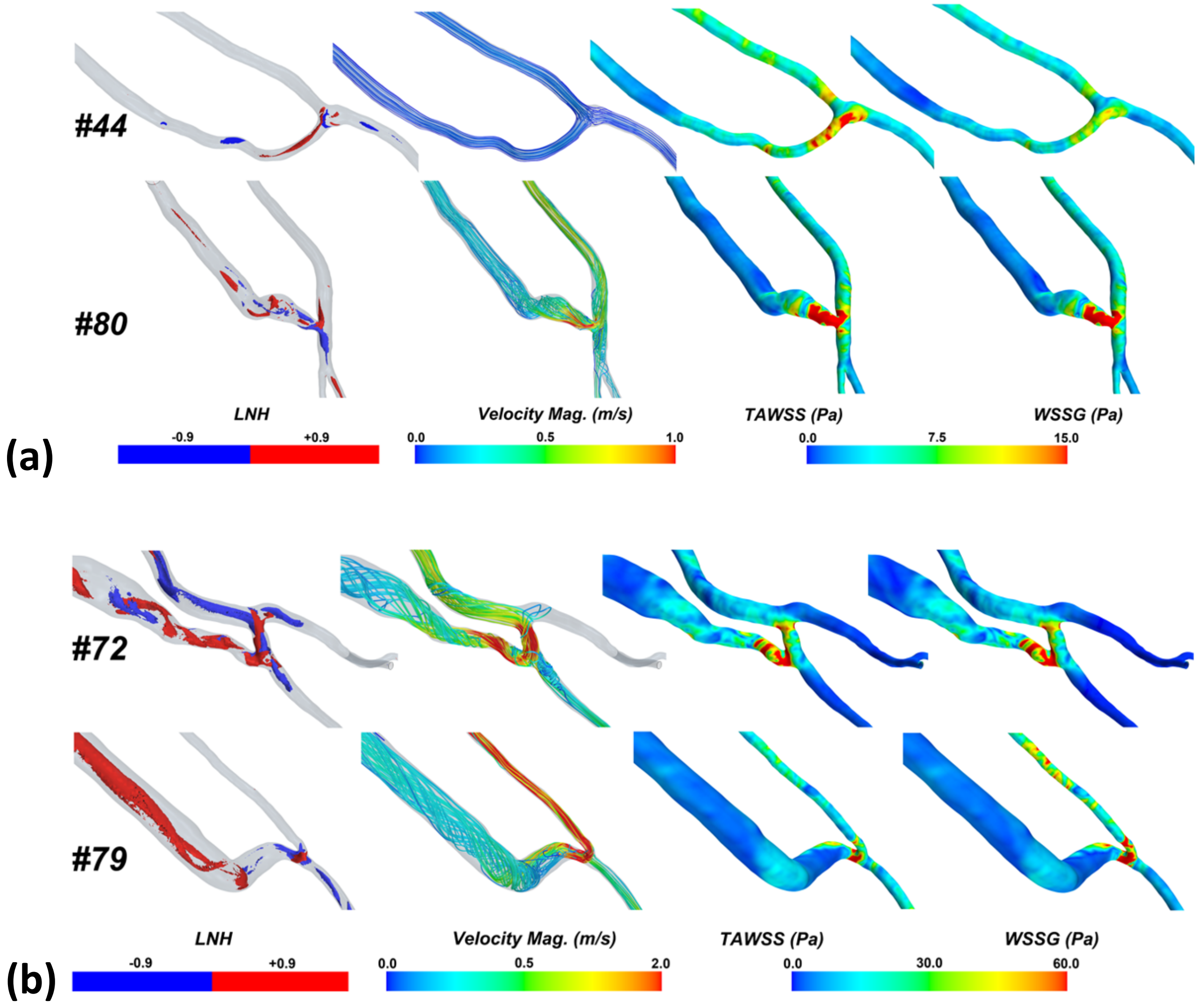

Supplementary Figure 3. NLH and flow streamlines (at peak systole), TAWSS and WWSG contour plots for unsuccessful (a) BCFs, (b) RCFs.
